# Bibliometric analysis and description of research trends on nutritional management in Alzheimer’s disease patients (1988–2024)

**DOI:** 10.3389/fnut.2025.1545951

**Published:** 2025-03-28

**Authors:** Chenchen Meng, Bei Li, Zhaoxia Wang, Qing Peng

**Affiliations:** Neurology Department, Peking University First Hospital, Beijing, China

**Keywords:** bibliometrics, publication, Alzheimer’s disease, nutritional management, diet

## Abstract

**Background:**

Alzheimer’s disease (AD) is a severe neurodegenerative disorder. Nutritional management has been recognized as a potential therapeutic approach to mitigate AD progression. This study aimed to analyze the bibliometric characteristics and research trends of publications on nutritional management in AD.

**Methods:**

A systematic search was conducted on the Web of Science Core Collection database to identify publications related to nutritional management in AD from 1988 to 2024. Bibliometric analysis was performed using VOSviewers (V 1.6.20), CiteSpace (V 6.3.R1) and R 4.3.3.

**Results:**

A total of 554 publications from 311 countries led by the USA were identified. The number of publications has increased annually. The most cited article discussed the role of diet in providing antioxidants to combat oxidative stress in neurodegenerative diseases. The University of California system published the most articles, and Rush University had the most international collaborations. These publications came from 3,298 authors, among which Mattson MP had the highest total publications and citations. The *Journal of Alzheimer’s Disease* published the most articles and received the most citations. Keywords analysis revealed evolving trends, with early emphasis on “precursor protein” and later shifts to “dietary restriction,” and more recently, “insulin resistance” and “synaptic plasticity.” Emerging keywords include “obesity,” “cognitive impairment” and “association.”

**Conclusion:**

This study represents the first summary of research trends in AD nutritional management. Future research is likely to focus on the associations between nutrition, insulin resistance, synaptic plasticity, and cognitive impairment in AD patients. This information provides valuable insights for scholars and practitioners in the field.

## Introduction

Alzheimer’s disease (AD) is a chronic neurodegenerative disorder characterized by cognitive decline, memory impairment, and behavioral disturbances, affecting a significant portion of the elderly population worldwide (1, 2). The disease is associated with the progressive loss of neurons and synaptic connections in the brain, leading to severe impairment in daily living activities (3, 4). Currently, the management of AD primarily mainly involves pharmacological interventions, such as cholinesterase inhibitors and memantine. These medications generally can only temporarily alleviate symptoms and so far have not been proven to halt or reverse the disease progression (5). Given the limitations of existing treatments, nutritional management has emerged as a potentially promising alternative approach to support AD patients (6). Nutritional management in AD involves the optimization of dietary intake to address nutrient deficiencies, reduce oxidative stress, and support brain health (7, 8). Dietary interventions, such as the Mediterranean diet, ketogenic diet, and omega-3 fatty acid supplementation, have shown potential benefits in slowing cognitive decline and improving overall quality of life in AD patients (9, 10). However, the effectiveness and optimal implementation of these interventions remain uncertain, necessitating further research.

Bibliometrics is a quantitative and qualitative method for assessing the output and impact of publications within a specific research field (11). By analyzing publication trends, citation patterns, and keyword frequencies, bibliometric analysis can provide insights into the evolution of research themes, identify key researchers and institutions, and assess the impact of various studies (12). Xu et al. elucidated the research status, frontiers, and trends of diet’s role in Alzheimer’s disease (2003–2023) via CiteSpace, suggesting future trends will focus on dietary patterns’ effectiveness and mechanisms using biomarkers and nutrient supplements (13). However, to our knowledge, no bibliometric analysis has been conducted specifically on nutritional management in AD patients. Therefore, this study aimed to perform a bibliometric analysis of publications on nutritional management in AD patients from 1988 to 2024. By analyzing the relevant literature, we aimed to identify major contributors, current research status, and emerging trends in this field. This analysis will contribute to a better understanding of nutritional management strategies for AD patients and facilitate the development of more effective interventions.

## Materials and methods

### Search strategies and data collection

We conducted a literature search on the Web of Science Core Collection (WoSCC) on nutritional management in AD (from 1988 to 2024). The search formula is (((TS = (Alzheimer Disease)) OR TS = (Alzheimer Syndrome)) OR TS = (Alzheimer Dementia)) AND (((((TS = (Nutritional Management)) OR TS = (Diet Therapy)) OR TS = (Dietary Modification)) OR TS = (Diet Modification)) OR TS = (Dietary Restriction)) (14–16). In all the literature searched, only the English article was included in this study for subsequent analysis. To avoid bias in database updates, we conducted the literature search on 22 June 2024. All information is collected in text form, including publication and citation counts, titles, author information, institutions, countries/regions, keywords, journals and other information for bibliometric analysis.

### Statistical analysis and visualization

Relevant data were extracted from the retrieved bibliographic records. Bibliometric indicators were identified and calculated by Microsoft Excel. Visualization analysis employed VOSviewer (V 1.6.20), CiteSpace (V 6.3.R1), and R 4.3.3 to conduct a comprehensive analysis of academic data.

VOSviewer mapped and visualized the co-occurrence of countries, institutions, authors and keywords within the selected literature. The results revealed main research themes and collaborations in the field (17). The network map featured nodes for countries, institutions, authors and keywords, with node size reflecting frequency of occurrence and color indicating different research clusters (or average publication year when analyzing keywords). Thicker lines between nodes denoted stronger co-occurrence relationship (18). CiteSpace analyzed the temporal evolution of research trends, generating time-zone maps to trace the historical development and emerging trends in nutritional management in AD (19). The resulting maps showed clusters of research topics, with cluster size indicating significance and color gradient showing the time span of research activities (20). R 4.3.3 was used for advanced statistical analysis and calculation of bibliometric indices (21). It enabled us to determine the H-index, G-index, and M-index for top authors and institutions. The H-index, or Hirsch index, assesses productivity and citation impact. The G-index emphasizes higher citation counts across a broader range, while the M-index balances paper production and citations received (12, 22). In this study, authors’ H-indices were sourced directly from the WoSCC database.

## Results

### An overview of publications in research of nutritional management in AD

**Figure 1:**
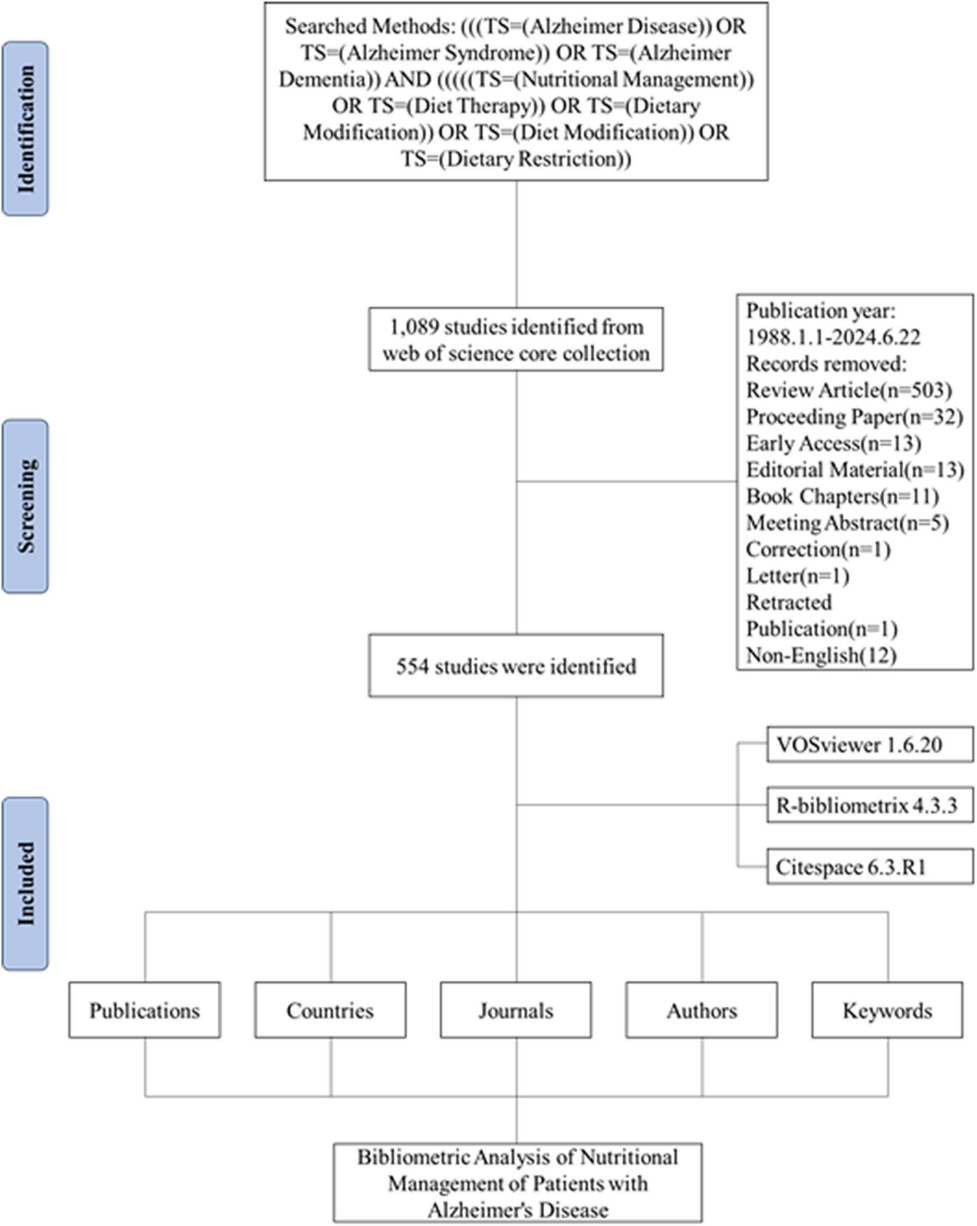
Flow diagram of the bibliographic retrieval process.

**Figure 2:**
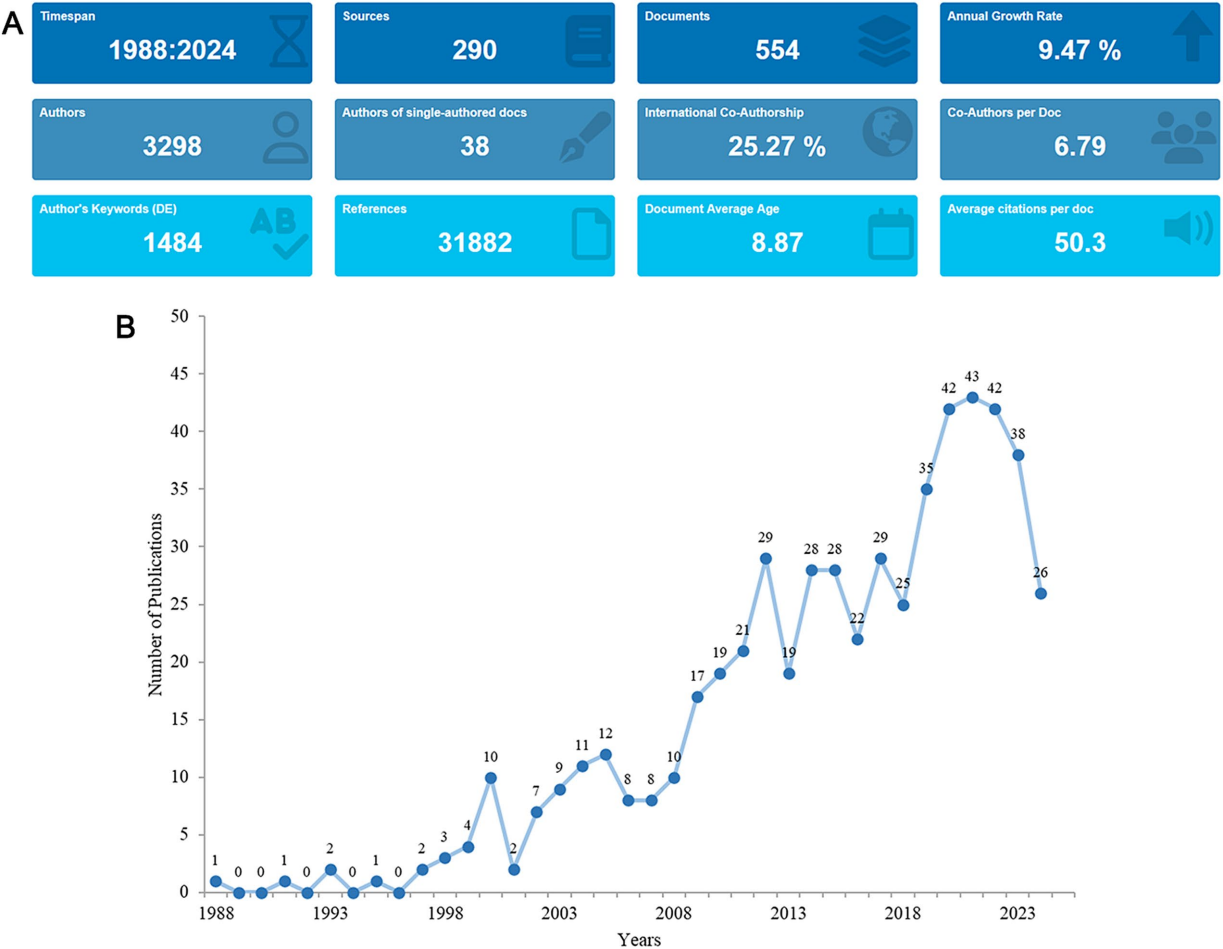
Analysis of the general information. **(A)** A summary of quantitative analysis of the publications. **(B)** Annual output of research from 1988 to 2024.

From the search strategy, the total number of publications concerning the research theme of nutritional management in AD published between 1988 and 2024 were 1,089. After exclusion, 554 English articles were identified, from which, information such as countries, journals, authors and keywords were extracted (Figure 1). A total of 3,298 authors from 2,180 institutions spanning 311 countries and regions participated in the production process. Their works were dispersed across 290 journals, referencing 31,882 sources in total. On average, each piece of literature had 6.79 co-authors, marking an international co-authorship rate of 25.27%. Furthermore, these publications encompassed 1,484 author keywords and received an average citation count of 50.3 per literature (Figure 2A).

Between 1988 and 2024, the annual publication volume of relevant literature exhibited a fluctuating upward trend, with an annual growth rate of 9.47%. In the early years (1988–1999), the publication fluctuated between 0 and 4 articles. The first peak appeared in 2000 (*n* = 10), followed by a sharp decline to 2 articles in 2001. The second and third peaks emerged in 2005 (*n* = 12) and 2012 (*n* = 29), respectively. During the period from 2012 to 2018 (*n* = 25), the publication volume remained relatively stable. Subsequently, the publication volume surged from 35 in 2019 to 43 in 2021. Since 2022, there has been a gradual decline (Figure 2B).

Among the top 50 most cited literatures (Supplementary Table S1), the article titled “*Oxidative Stress and Neurodegenerative Diseases: A review of upstream and downstream antioxidant therapeutic options*” was published in the *Current Neuropharmacology* (IF 2023 = 4.8) in 2009 and accumulated the most cited count of 2,376 citations. The article reviewed that diet plays a crucial role in providing antioxidants, which help combat oxidative stress and free radicals, thereby protecting neural cells and preventing neurodegenerative diseases and other disorders (23). In addition, the article published in the *Nature Genetics* (IF 2023 = 31.7) in 2000, titled “*Gene-expression profile of the aging brain in mice*” has garnered a total of 858 citations (24). The article titled “*Folic acid deficiency and homocysteine impair DNA repair in hippocampal neurons and sensitize them to amyloid toxicity in experimental models of Alzheimer’s disease*” published in the *Journal of neuroscience* (IF 2023 = 4.4) in 2002 accumulated a total of 531 citations (25).

### Distribution and collaborative networks of countries

**Figure 3:**
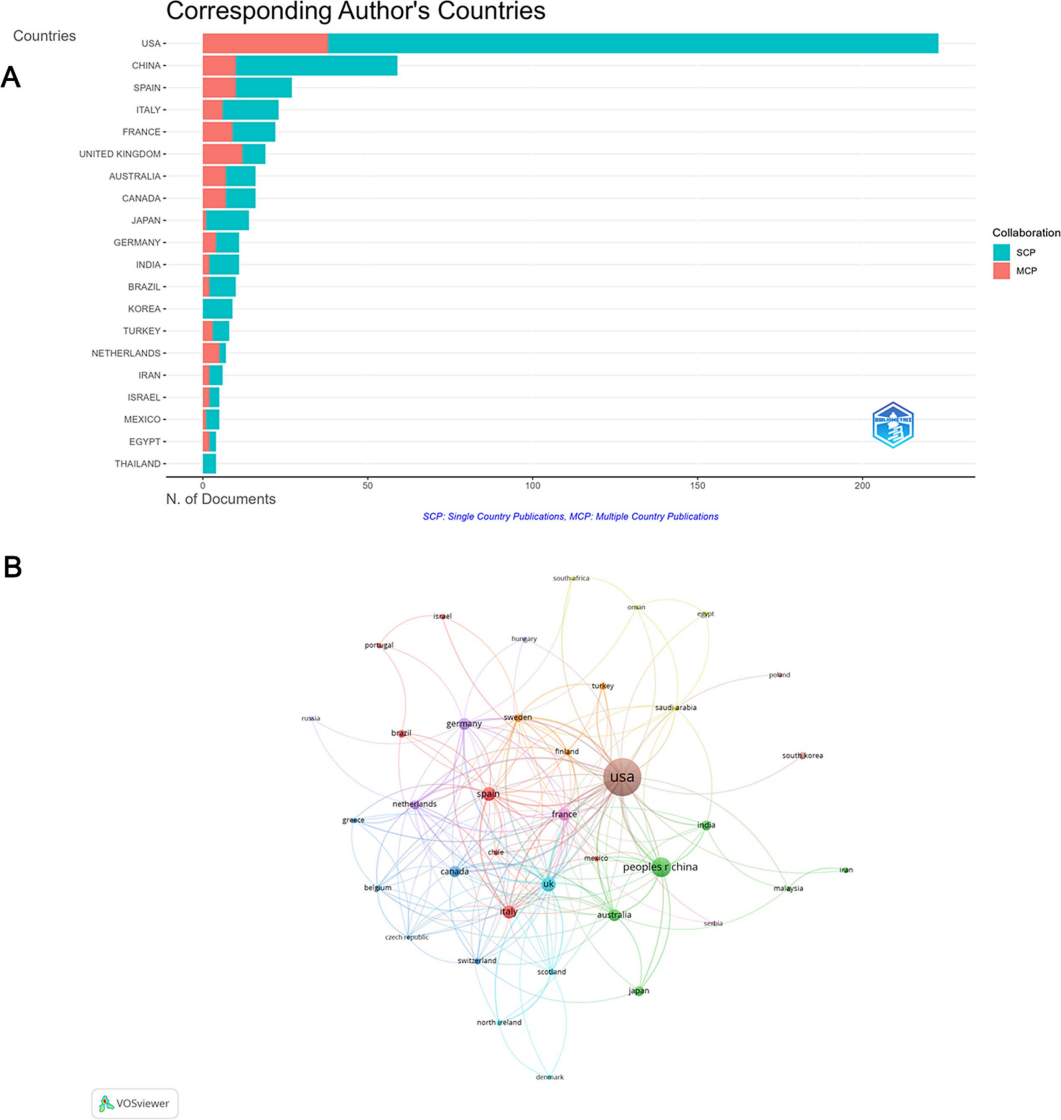
Analysis of countries. **(A)** Distribution of responding author’s publications by country. **(B)** Visualization map depicting collaboration among different countries.

These publications were from 311 countries, 39 among which were involved in international collaborations with a minimum of 3 articles. Among the countries, the country with the largest number of articles is the USA (*n* = 223, 40.3%), followed by China (*n* = 59, 10.6%) and Spain (*n* = 27, 4.9%). The combined number of articles from the USA and China accounted for over half of the total (50.9%) (Figure 3A; Supplementary Table S2). Furthermore, the USA topped the number of collaborations, manly with China, the UK, France, Canada and Sweden. The UK ranked 2nd in international collaborations, having active cooperation with Sweden, Germany and Netherlands (Figure 3B).

### Distribution and collaborative networks of institutions

**Figure 4:**
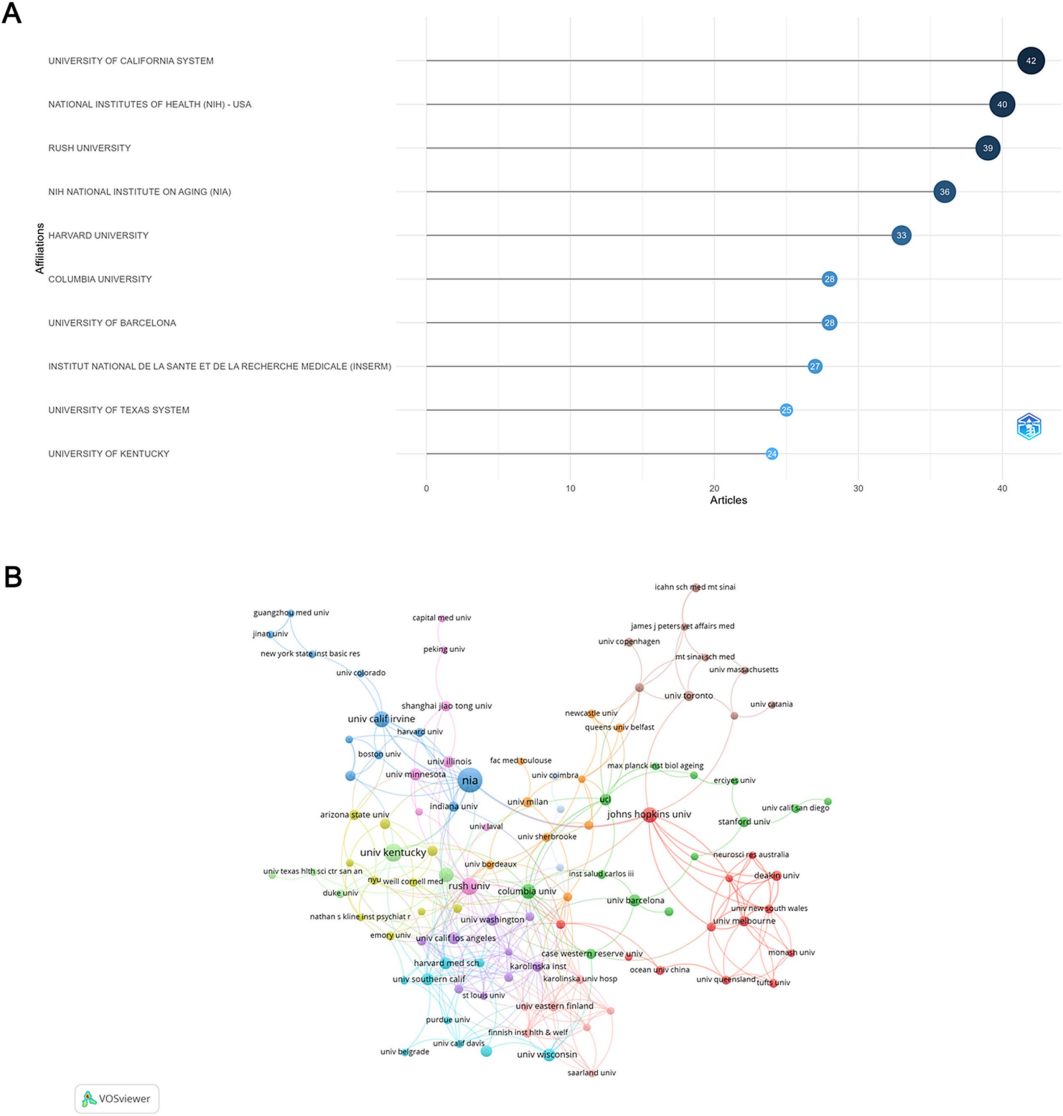
Analysis of institutions. **(A)** Top 10 institutions ranked by article count. **(B)** Visualization map depicting collaboration among different institutions.

A total of 2,180 institutions participated in the publication of relevant articles and 123 among them were involved in international collaborations with a minimum of 3 articles. Among the top 10 institutions ranked by article count (Figure 4A), University of California System stood out with the most publications (TP = 42), followed by National Institute of Health (NIH) (TP = 40) and Rush University (TP = 39), all from the USA. Despite ranking 3rd in terms of publication volume, Rush University had the highest number of international collaborations, tying with the University of Eastern Finland, followed by Karolinska Institute in Sweden (Figure 4B).

### Authors and co-authors analysis

**Figure 5:**
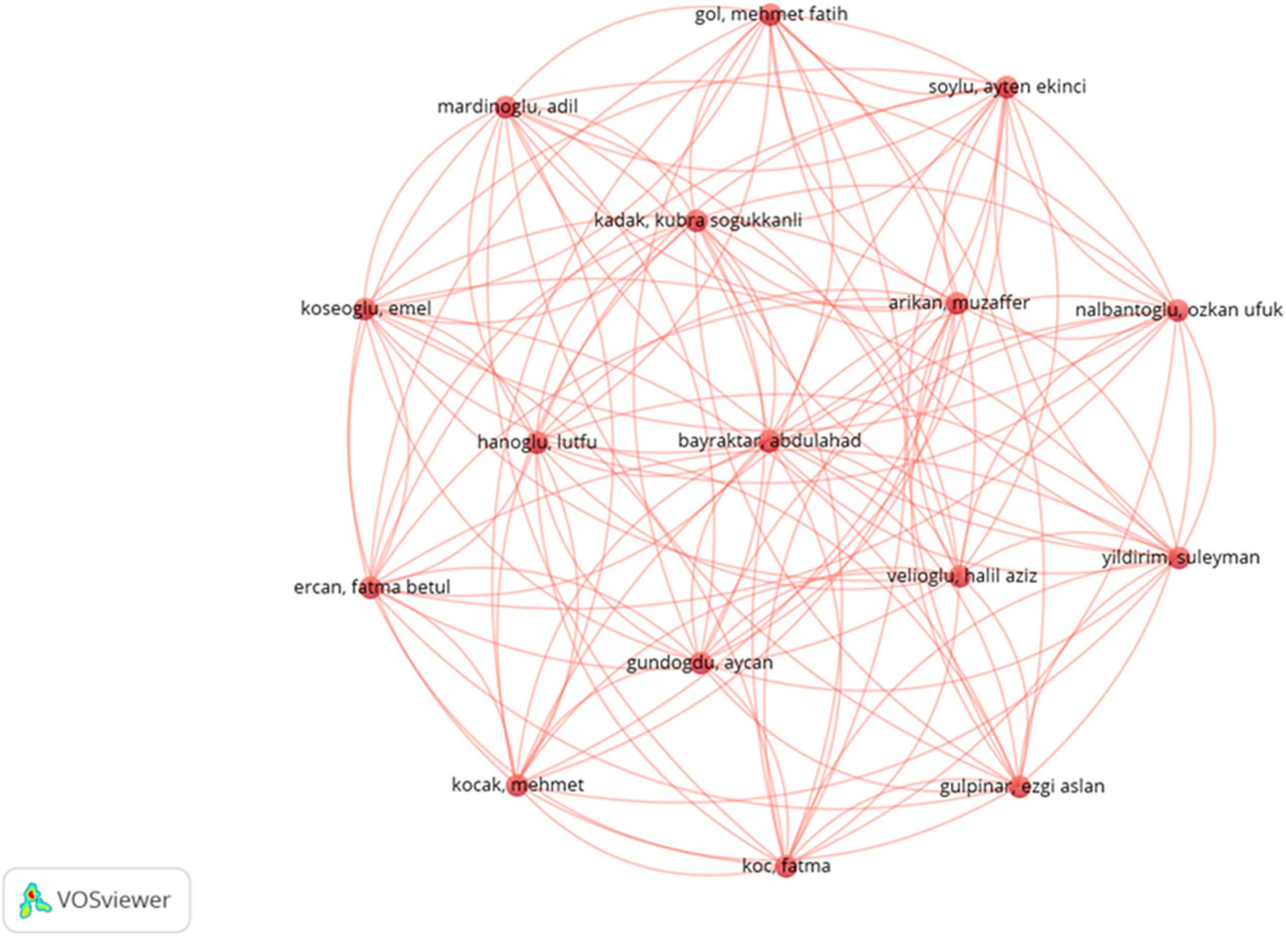
The visualization map depicting collaboration among authors.

A total of 3,298 authors participated in research of nutritional management in AD and 236 authors were involved in international collaborations with a minimum of 2 articles. Mattson MP topped the lists with the highest total publications (TP = 27) and most total cited counts (TC = 4,469) (Supplementary Table S3). The H-index, G-index and M-index of the author were 25, 27 and 0.962, respectively. The most highly cited paper by the author is the aforementioned “*Folic Acid Deficiency and Homocysteine Impair DNA Repair in Hippocampal Neurons and Sensitize Them to Amyloid Toxicity in Experimental Models of Alzheimer’s Disease*” with 531 citations. This study indicated that folic acid deficiency and elevated homocysteine levels impair DNA repair in neurons, thereby increasing their vulnerability to amyloid *β*-peptide toxicity and potentially contributing to the risk of AD (25). Furthermore, Vellas B ranked 2nd in total publications (TP = 10) and 3rd in total citations (TC = 780). Andrieu S ranked 3rd in total publications (TP = 8), while Lee J ranked 2nd in total citations (TC = 970). In terms of collaboration, a total of 16 authors rank 1st in collaboration intensity (see details in Figure 5).

### Contributions and collaborative networks of journals

**Figure 6:**
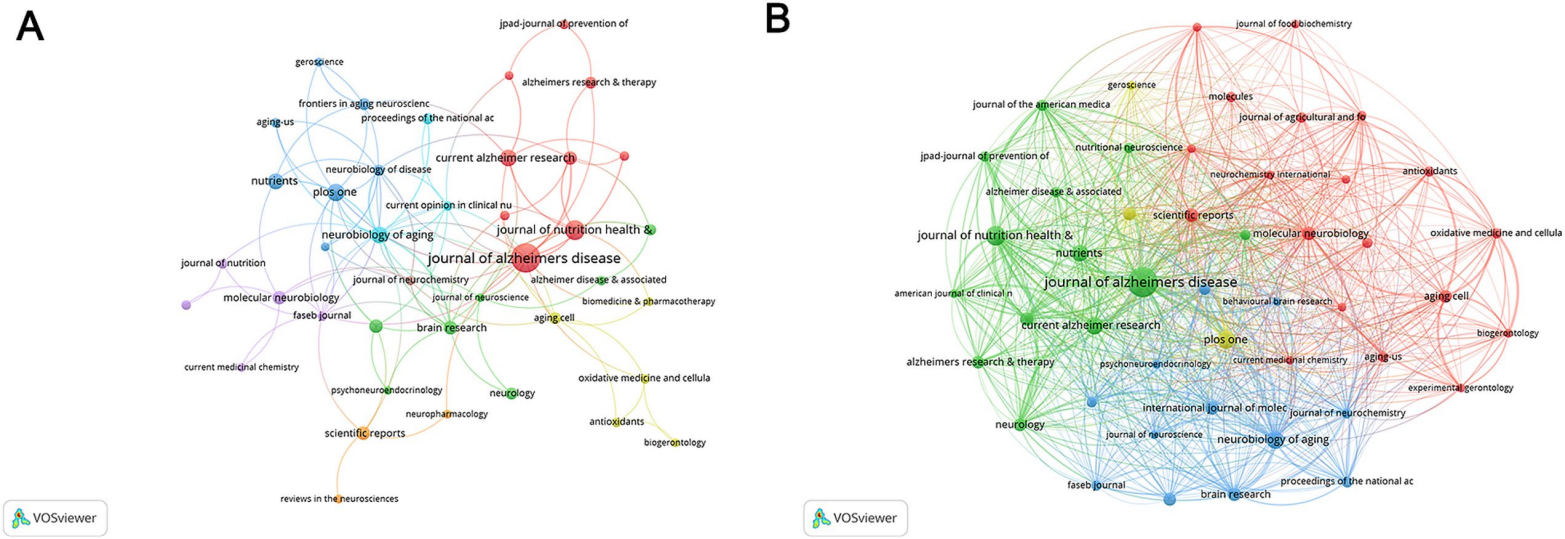
Analysis of journals. **(A)** The co-occurrence networks of journals. **(B)** The coupling networks of journals.

Publications related to nutritional management in AD were published in 290 journals, containing 47 with at least 3 occurrences and 30 with at least 3 couples. *Journal of Alzheimer’s disease* (IF 2023 = 3.4, H-index = 16) achieved the most total publication counts (TP = 34) and most total cited counts (TC = 842) (Supplementary Table S4). As shown in Figures 6A,B, this journal ranked 2nd in co-occurrence networks and 1st in coupling networks. *Journal of nutrition health & aging* (IF 2023 = 4.3, H-index = 12) ranked 2nd in total publication counts (TP = 15), and 3rd in coupling networks. The Journal *Proceedings of the national academy of sciences of the United States of America* ranked 2nd in total cited counts (TC = 792) with only 5 publications. Besides, the *Neurobiology of aging* ranked 1st in co-occurrence networks. The *Neurobiology of disease* ranked 3rd in co-occurrence networks and 2nd in coupling networks.

### Keyword co-existence network and burst keywords

**Figure 7:**
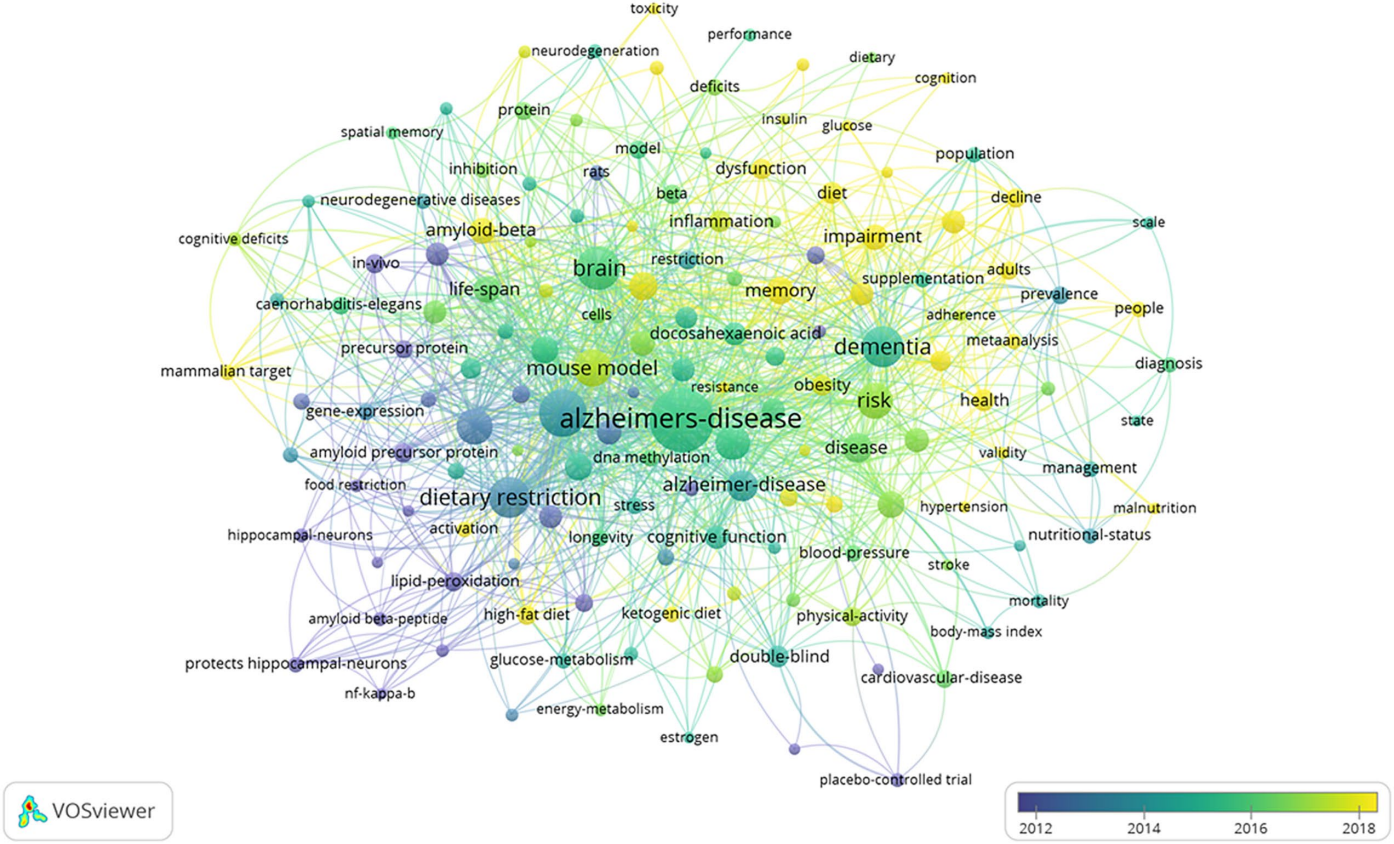
Visual analysis of keyword co-occurrence network.

In total, 150 keywords with a minimum of 6 occurrences were identified (Figure 7). “Alzheimer’s disease” appears the most frequently, followed by “oxidative stress” and “brain.” The frequencies of “dietary restriction,” “dementia” and “mouse model” followed closely behind. Additionally, other keywords related to dietary intake such as “caloric restriction,” “Mediterranean diet” and “a-beta” also appeared with relatively high frequencies.

**Figure 8:**
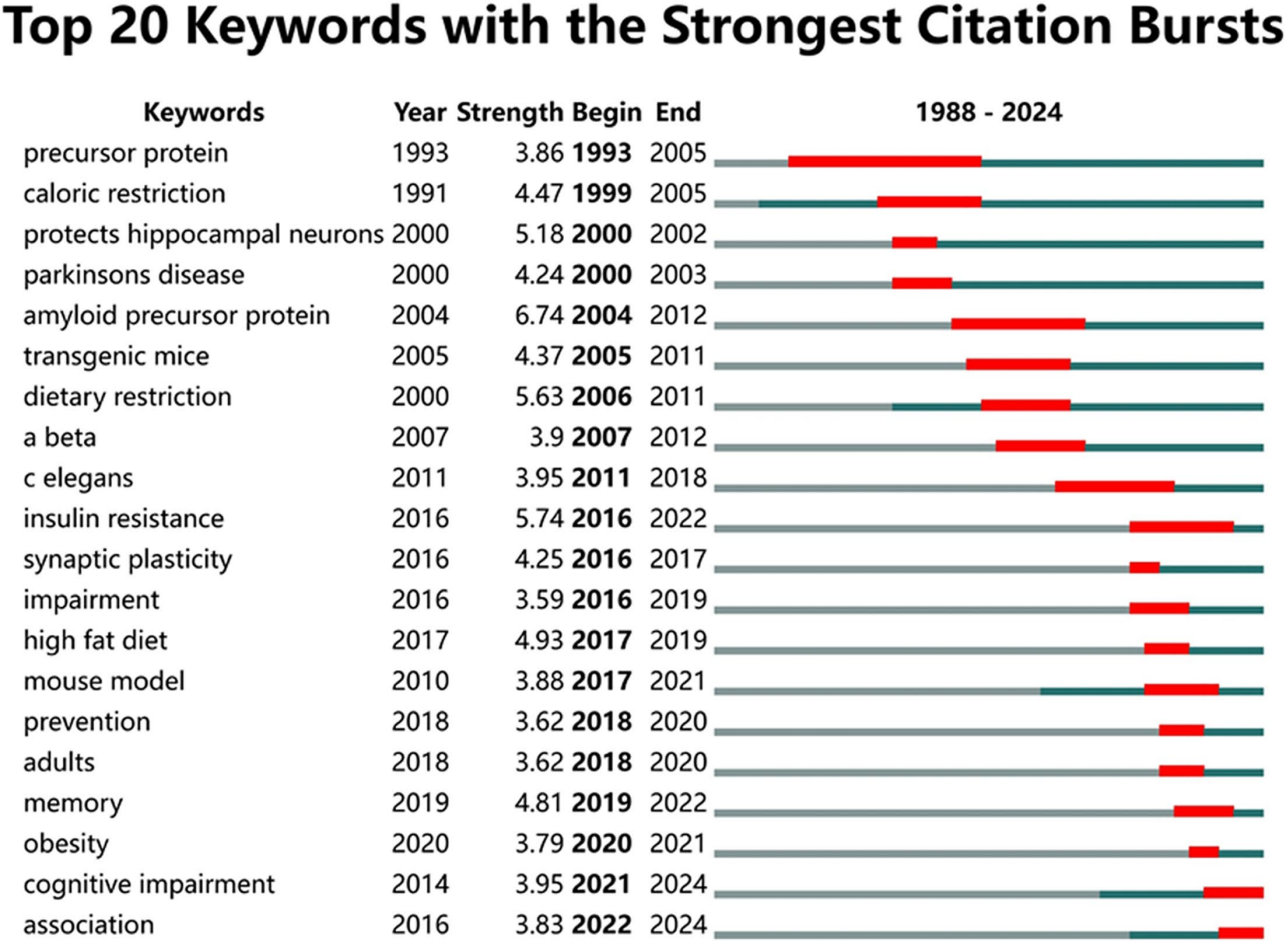
Top 20 keywords with the strongest citation bursts.

The burst analysis of keywords spanning from 1988 to 2024 was conducted to uncover evolutionary trends. The top 20 keywords with the strongest citation bursts during the specified period were listed in Figure 8. The keyword “precursor protein” (strength 3.86) emerged as the earliest and had the longest duration of burst (1993–2005). Subsequently, “caloric restriction” (strength 4.47, 1999–2005) and “protects hippocampal neurons” (strength 5.18, 2000–2002) appeared. The most significant citation burst belonged to “amyloid precursor protein” (strength 6.74, 2004–2012). Between 2005 and 2011, “transgenic mice” (strength 4.37, 2005–2011), “dietary restriction” (strength 5.63, 2006–2011), “a-beta” (strength 3.9, 2007–2012) and “c elegans” (strength 3.95, 2011–2018) experienced bursts successively. From 2016 onwards, “insulin resistance” (strength 5.74, 2016–2022), “synaptic plasticity” (4.25, 2016–2017), “high-fat diet” (strength 4.93, 2017–2019) and “prevention” (strength 3.62, 2018–2020) emerged as burst keywords. In recent years, “obesity” (strength 3.79, 2020–2021), “cognitive impairment” (strength 3.95, 2021–2024) and “association” (strength 3.83, 2022–2024) have seen outbreaks.

## Discussion

The present bibliometric study revealed a total of 554 publications on nutritional management in AD patients from 1988 to 2024. These publications were contributed by 3,298 authors from 2,180 institutions across 311 countries, with a notable international collaboration rate of 25.27%. The research output distributed across 290 journals demonstrated a steady increase in annual publication volume, peaking in recent years. The top-cited articles reflected a growing interest in nutritional interventions. The trends of keywords revealed a progression from basic biological mechanisms to complex nutritional interventions and their impact on cognitive outcomes.

The field of nutritional management in AD gained momentum post-2000, with significant spikes around 2005 and 2012. This pattern may reflect advancements in understanding AD pathophysiology and the recognition of nutritional interventions as one of potential therapeutic strategies (26). The recent surge, starting from 2019, could be attributed to emerging research on diet-gene interactions, the role of metabolites in AD progression, and the development of novel nutritional interventions (27). The emergence of high-impact articles highlighted the significance of antioxidants, gene expression profiles, and dietary deficiencies in AD progression. They also indicated key turning points in research, such as the recognition of oxidative stress as a critical factor in neurodegeneration (23). Through the quality assessment of these highly cited articles, it is evident that while they have significantly contributed to our understanding of AD’s nutritional management and pathophysiology, they each exhibit certain limitations in research design. First, many existing studies rely on animal models such as transgenic mice or *C. elegans* (28, 29). While these models are invaluable for exploring molecular mechanisms, their metabolic and physiological differences from humans limit the direct translation of findings into clinical practice (26). Second, numerous clinical studies suffer from small sample sizes and lack long-term follow-up, making it difficult to evaluate the sustained effects of specific dietary interventions on cognitive function (30). Future research should prioritize large-scale, multicenter randomized controlled trials (RCTs) to enhance the robustness and applicability of findings. Additionally, although many studies have examined the effects of individual nutrients or dietary components, there is a lack of comprehensive investigations into how overall dietary patterns (e.g., the Mediterranean diet or ketogenic diet) interact with genetic factors, gut microbiota, and metabolic pathways (31). A more integrative and multi-layered research approach is required to advance precision nutrition strategies tailored to AD patients. Moreover, to ensure the reliability of future clinical trials, rigorous methodological frameworks must be implemented. Several studies have highlighted key strategies to improve trial design, such as incorporating stratified randomization based on genetic predispositions (e.g., APOE ε4 carriers), metabolic profiles, and pre-existing dietary habits to enhance the relevance of findings to diverse populations (32). Additionally, the inclusion of standardized dietary assessment tools and biomarkers—such as cerebrospinal fluid (CSF) tau/Aβ ratios and inflammatory markers—has been recommended to objectively measure intervention efficacy (33). Longitudinal studies with extended follow-up durations are crucial for evaluating long-term cognitive and metabolic outcomes, as short-term trials may fail to capture the full therapeutic potential of dietary interventions (34). By adopting these methodological improvements, future trials can generate more conclusive evidence on the role of dietary interventions in AD prevention and management. The leading contributions from the USA, China, and Spain reflect their substantial investments in AD research and development. The United States leads in the number of studies in this field, particularly focusing on the impact of oxidative stress and gene expression on AD (35). This trend may be attributed to the substantial funding provided by the National Institutes of Health (NIH) for neurodegenerative disease research, as well as the long-standing focus on AD pathophysiology in Western countries. Oxidative stress is considered a key factor in AD pathology, as it promotes *β*-amyloid (Aβ) aggregation, mitochondrial dysfunction, and neuroinflammation, thereby accelerating cognitive decline (23). Additionally, the United States’ leading position in genomics and bioinformatics has enabled researchers to focus on gene–environment interactions in neurodegenerative diseases (36). In particular, American studies emphasize the role of dietary antioxidants, such as polyphenols, vitamins E and C, in alleviating oxidative stress and mitigating cognitive decline (9). In contrast, China has witnessed rapid growth in AD nutritional management research in recent years, with a strong emphasis on the neuroprotective effects of traditional Chinese medicine (TCM) and plant-derived bioactive compounds (11). This research trend is closely linked to China’s long-standing emphasis on herbal medicine. For instance, flavonoids, ginsenosides, and resveratrol have been shown to reduce Aβ toxicity, inhibit tau protein phosphorylation, and improve synaptic plasticity in *in vitro* and animal models (37). Furthermore, the Chinese government’s policy support for “integrative medicine, “along with a rapidly aging population, has led to increased funding for AD treatment research based on traditional medicine (31). However, most TCM-related studies remain at the cellular and animal experiment stage, with a lack of large-scale RCTs to validate their long-term clinical efficacy. Additionally, research in Spain has primarily focused on the relationship between the Mediterranean diet and AD, particularly regarding obesity, metabolic syndrome, and cognitive decline (30). The Mediterranean diet is rich in antioxidants, polyphenols, unsaturated fatty acids, and dietary fiber, and studies have shown that this dietary pattern can improve insulin sensitivity, reduce chronic inflammation, and lower AD risk (9). Researchers in Spain and other Mediterranean countries have placed particular emphasis on how dietary patterns influence neuroinflammation and metabolic function through the gut-brain axis, thereby modulating AD pathogenesis (38). However, despite the observed regional trends, there remains a lack of systematic analysis of how different dietary patterns specifically affect AD pathogenesis. Future studies should integrate multi-omics approaches (genomics, metabolomics, and microbiomics) to explore the comprehensive effects of these dietary patterns on neurological health. Additionally, large-scale, multicenter clinical studies are needed to validate their applicability across different populations (39). The dominance of American institutions like the University of California system and the NIH underscores their significant role in driving research forward. However, despite the high publication output, some of the institutions had limited collaborations, which could potentially hinder comprehensive progress in this research field. Therefore, fostering broader and deeper international collaborations among institutions could significantly propel the development of nutritional management strategies for Alzheimer’s disease patients. Mattson MP stood out as the most prolific author, contributing significantly to our understanding of the role of oxidative stress and nutrition in AD. His research on the role of diet in DNA repair and amyloid toxicity exemplifies the depth of investigation into nutritional management strategies (25). The collaboration intensity among top authors indicates a collaborative research culture that fosters innovation and progress. The concentration of publications in journals like the *Journal of Alzheimer’s Disease* and *Journal of Nutrition, Health & Aging* indicates their dedication to AD research. The preference for certain journals might be due to their focus on neuroscience and aging-related research, aligning well with the AD nutrition management theme. Journals with a strong emphasis on aging and neurodegenerative diseases tend to attract submissions on AD nutrition, reflecting their alignment with the journal’s scope and readership interests.

The keyword analysis in this study provides a nuanced understanding of the evolving research landscape on nutritional management in AD patients. The appearance of “Alzheimer’s disease” as the most frequent keyword underscores its central focus, with “oxidative stress” and “brain” closely following, indicating a robust emphasis on the mechanisms underlying neuronal damage in AD. This alignment with extensive literature emphasizes the pivotal role of oxidative stress in AD pathogenesis, confirming its critical involvement in neuronal degeneration (35).

The emergence of keywords like “dietary restriction,” “dementia,” and “mouse model” highlights the significant role of nutritional interventions and animal studies in advancing AD research. These keywords reflect a concerted effort to investigate the impact of dietary modifications on AD progression, as evidenced by the frequent mentions of “caloric restriction” and “Mediterranean diet” (30, 40). The focus on these diets, particularly their neuroprotective properties, underscores the research community’s interest in exploring non-pharmacological interventions as alternative or complementary treatments for AD (31). This shift towards dietary modulation as a potential therapeutic strategy is particularly noteworthy, given the increasing recognition of the limitations and side effects associated with traditional pharmacological approaches.

The consistent mention of “a-beta” reveals a sustained focus on amyloid-beta peptide. Amyloid-beta is the core pathogenic substance in AD, with its deposition in the brain forming senile plaques, which is a major pathological change in AD. Researchers continue to delve into the role of amyloid-beta in neuronal dysfunction, aiming to develop strategies to mitigate its neurotoxic effects (37). This ongoing effort underscores the critical need for a deeper understanding of AD’s underlying mechanisms to facilitate the development of more effective treatments.

The burst analysis spanning from 1988 to 2024 provides a dynamic view of how research trends have evolved over time. Early bursts centered around “precursor protein,” the precursor to Amyloid-beta, which is a hallmark of AD pathology. This burst reflects an initial emphasis on elucidating the genetic and molecular underpinnings of AD (36). The emergence of “caloric restriction” and “protects hippocampal neurons” in the late 1990s and early 2000s marked a significant shift towards exploring the neuroprotective effects of dietary interventions. This shift was driven by the recognition that dietary factors could play a crucial role in preserving neuronal health and mitigating the progression of AD (41).

The period between 2005 and 2011 witnessed a surge in keywords like “transgenic mice,” “dietary restriction,” “a-beta,” and “c elegans,” marking a period of intense research into animal models and the mechanisms through which dietary factors influence AD pathology. The use of transgenic mice provided more precise models of AD, enabling researchers to investigate the effects of dietary interventions with greater accuracy (29). Studies in c elegans offered insights into conserved pathways of neurodegeneration across species, further enhancing our understanding of AD’s underlying mechanisms (28).

More recently, the focus on “insulin resistance” aligns with emerging evidence linking metabolic disturbances to AD risk. This research direction suggests that metabolic factors may play a significant role in AD pathogenesis and that interventions targeting these factors may offer therapeutic benefits (39). Similarly, the emergence of “synaptic plasticity” as a central theme reflects ongoing efforts to enhance cognitive resilience in AD patients (42). This research direction holds promise for developing interventions that can improve cognitive function and delay cognitive decline. The appearance of “high-fat diet” raises concerns about dietary patterns that may exacerbate AD risk, prompting investigations into preventive strategies (43). These recent trends underscore the importance of identifying modifiable risk factors and developing interventions that can delay or prevent cognitive decline.

The most recent bursts, featuring “obesity” “cognitive impairment” and “association,” reflect the evolving understanding of AD as a multifactorial disease with metabolic, cognitive, and lifestyle components intricately linked (38). These keywords suggest that research is increasingly focused on identifying modifiable risk factors and developing interventions that can delay or prevent cognitive decline. This research direction is crucial for advancing our understanding of AD and for developing effective interventions that can improve the quality of life for AD patients.

The timeline of keyword bursts highlights several key research bottlenecks and potential future directions. Early bursts focused heavily on basic biological mechanisms, while later bursts introduced more complex, translational research questions. However, despite significant advancements, the development of effective therapies targeting these pathways has remained challenging. For instance, while enhancing synaptic plasticity holds promise for cognitive enhancement, identifying safe and effective ways to do so remains a challenge (44). Similarly, understanding the role of insulin resistance in AD requires further clarification of its underlying mechanisms and potential interactions with other metabolic factors (45).

However, despite the significant progress in AD nutritional management research over the past decades, several critical gaps remain unresolved. First, most studies have been conducted primarily on Western populations, with limited data on other ethnic and geographic groups, restricting the generalizability of findings (38). For example, nearly all studies on the Mediterranean diet have been conducted in European populations, leaving its impact on Asian, American, and African populations unclear. Second, while research has identified a strong association between insulin resistance and AD risk, the causal relationship remains uncertain—whether insulin resistance directly contributes to AD pathogenesis or merely coexists as a correlated condition has yet to be determined. Another crucial issue is the limited translational application of findings related to synaptic plasticity. Although this mechanism is widely recognized as a key factor in cognitive function, current studies have yet to effectively translate these biological mechanisms into feasible clinical interventions (42). To address these issues, future research should enhance interdisciplinary collaboration, incorporate more diverse study populations, and adopt more rigorous research designs to ensure scientific robustness and broad applicability of findings. Additionally, systematic reviews and meta-analyses could further consolidate existing evidence and provide stronger empirical support for developing nutrition management strategies for AD patients.

Future research is likely to benefit from further exploring the association between dietary factors, metabolic disturbances, and cognitive decline in AD. There appears to be a pressing need for longitudinal studies that investigate the long-term effects of dietary interventions on AD progression. Additionally, studies that incorporate multidisciplinary approaches, including genetics, neuroscience, and nutrition, will be essential for advancing our understanding of AD’s complex etiology and developing more effective treatments. As the research community continues to unravel the mysteries of AD, the hope remains that dietary interventions will emerge as powerful tools in the fight against this devastating disease.

Although this study primarily focuses on bibliometric analysis of nutritional management in AD patients, we recognize that the clinical application of these interventions is a vital component of this research field. Bibliometric analysis offers valuable insights into emerging research trends and can guide the design of future clinical studies. By analyzing the current body of literature, we are able to identify promising areas for dietary interventions that could benefit AD patients and inform clinical practice as well as public health strategies. As research on dietary interventions continues to evolve, future clinical trials should explore the long-term effects of these interventions on AD patients. For example, the growing emphasis on oxidative stress and gene–environment interactions in AD research highlights the need for targeted clinical trials that evaluate antioxidant therapies or personalized treatment strategies. Similarly, the increasing focus on traditional Chinese medicine in AD treatment underscores the potential for integrating complementary therapies into mainstream clinical practice. While this study does not directly include clinical data, it underscores the importance of bridging the gap between research trends and clinical applications. To facilitate this, future research should incorporate clinical trial outcomes to validate the effectiveness of nutritional interventions. This will provide stronger evidence to support public health policies and improve patient care strategies in Alzheimer’s disease management.

### Strengths and limitations

The strengths of this bibliometric analysis lie in its comprehensive coverage of AD nutrition management research and the use of advanced metrics to capture research trends. Limitations include the exclusion of non-English publications and the reliance on citation metrics, which may not fully capture the impact of all research contributions. However, citation metrics offer a quantitative method to evaluate research influence. Given that non-English publications accounts for a relatively small proportion of the total included articles, we believe this has minimal influence on our overall analysis. Additionally, the analysis was limited to bibliometric data, lacking in-depth content analysis of research methodologies and findings. One more limitation is that the exclusive use of the WoSCC might miss out on relevant studies from other specialized or regional databases. In the future, we plan to expand our data sources to include databases like Scopus and PubMed to obtain a more comprehensive view of the research field. Despite the valuable insights provided by keyword co-occurrence analysis, this method has its limitations. Since it relies on keyword frequencies, it often misses the underlying complexities and connections between different research themes. To address this limitation, future studies could incorporate more detailed topic modeling analysis, such as Latent Dirichlet Allocation, which operates on abstracts or full texts to uncover more comprehensive and nuanced research trends. Topic modeling can help identify hidden topics, reveal shifting research interests, and highlight the relationships between different research areas over time (46).

## Conclusion

This bibliometric analysis reveals a dynamic and expanding research landscape on nutritional management in AD patients. The field has witnessed significant growth in publications, collaborations, and citations, with the United States leading the way. Research trends indicate a shift from foundational studies on amyloid precursor proteins to more recent explorations of dietary interventions, insulin resistance, and synaptic plasticity. The keyword analysis underscores the evolving nature of research interests, with obesity, cognitive impairment, and associations with AD risk factors emerging as new areas of focus. Ongoing bibliometric analyses are essential for monitoring research progress and guiding future studies, ensuring that the field continues to evolve in response to emerging scientific evidence and clinical needs. As the global population ages and AD incidence rises, the importance of nutritional management in AD prevention and management is likely to grow significantly, necessitating continued research and collaboration to advance our understanding and improve patient outcomes.
