# Supplementary material for: Bibliometric analysis and description of research trends on nutritional management in Alzheimer’s disease patients (1988–2024)
Source: Front Nutr. 2025 Mar 28;12:1545951. doi: 10.3389/fnut.2025.1545951 (PMC11985443; doi:10.3389/fnut.2025.1545951)
Supplement: Supplementary file 1 [file Table_1.docx]

**Table S1.** Top 50 articles with the highest cited counts.

| **Paper** | **DOI** | **TC** | **TC**  **per Year** | **Normalized TC** |
| --- | --- | --- | --- | --- |
| UTTARA B, 2009, CURR NEUROPHARMACOL | 10.2174/157015909787602823 | 2376 | 148.5 | 13.02 |
| LEE CK, 2000, NAT GENET | 10.1038/77046 | 858 | 34.32 | 5.04 |
| KRUMAN II, 2002, J NEUROSCI | 10.1523/JNEUROSCI.22-05-01752.2002 | 531 | 23.09 | 3.31 |
| VINGTDEUX V, 2010, J BIOL CHEM | 10.1074/jbc.M109.060061 | 518 | 34.53 | 5.93 |
| MATTSON MP, 2008, ANN NY ACAD SCI | 10.1196/annals.1418.005 | 444 | 26.12 | 4.42 |
| HALAGAPPA VKM, 2007, NEUROBIOL DIS | 10.1016/j.nbd.2006.12.019 | 411 | 22.83 | 3.82 |
| KAPOGIANNIS D, 2011, LANCET NEUROL | 10.1016/S1474-4422(10)70277-5 | 406 | 29 | 4.72 |
| XU W, 2015, J NEUROL NEUROSUR PS | 10.1136/jnnp-2015-310548 | 377 | 37.7 | 7.42 |
| LUCHSINGER JA, 2002, ARCH NEUROL-CHICAGO | 10.1001/archneur.59.8.1258 | 347 | 15.09 | 2.16 |
| BRUCE-KELLER AJ, 1999, ANN NEUROL | 10.1002/1531-8249(199901)45:1<8::AID-ART4>3.0.CO;2-V | 328 | 12.62 | 1.61 |
| NAGPAL R, 2019, EBIOMEDICINE | 10.1016/j.ebiom.2019.08.032 | 314 | 52.33 | 7.02 |
| LEE J, 2000, J MOL NEUROSCI | 10.1385/JMN:15:2:99 | 308 | 12.32 | 1.81 |
| TAYLOR RC, 2011, CSH PERSPECT BIOL | 10.1101/cshperspect.a004440 | 307 | 21.93 | 3.57 |
| MATTSON MP, 1999, ANN NY ACAD SCI | 10.1111/j.1749-6632.1999.tb07824.x | 288 | 11.08 | 1.42 |
| GREEN KN, 2007, J NEUROSCI | 10.1523/JNEUROSCI.0055-07.2007 | 287 | 15.94 | 2.67 |
| KIVIPELTO M, 2013, ALZHEIMERS DEMENT | 10.1016/j.jalz.2012.09.012 | 280 | 23.33 | 4.02 |
| ROMERO FJ, 1998, ENVIRON HEALTH PERSP | 10.2307/3433990 | 275 | 10.19 | 2.64 |
| PATEL NV, 2005, NEUROBIOL AGING | 10.1016/j.neurobiolaging.2004.09.014 | 274 | 13.7 | 2.69 |
| SUNG S, 2004, FASEB J | 10.1096/fj.03-0961fje | 262 | 12.48 | 3.1 |
| ERSHLER WB, 1993, LYMPHOKINE CYTOK RES | NA | 255 | 7.97 | 1.67 |
| SUN AY, 2010, MOL NEUROBIOL | 10.1007/s12035-010-8111-y | 253 | 16.87 | 2.89 |
| GERSTBREIN B, 2005, AGING CELL | 10.1111/j.1474-9726.2005.00153.x | 223 | 11.15 | 2.19 |
| WANG J, 2005, FASEB J | 10.1096/fj.04-3182fje | 217 | 10.85 | 2.13 |
| SCHELTENS P, 2010, ALZHEIMERS DEMENT | 10.1016/j.jalz.2009.10.003 | 210 | 14 | 2.4 |
| SCHELTENS P, 2012, J ALZHEIMERS DIS | 10.3233/JAD-2012-121189 | 208 | 16 | 4.49 |
| PORQUET D, 2013, AGE | 10.1007/s11357-012-9489-4 | 203 | 16.92 | 2.92 |
| MORLEY JE, 2015, J AM MED DIR ASSOC | 10.1016/j.jamda.2015.06.017 | 199 | 19.9 | 3.92 |
| HYUN DH, 2006, P NATL ACAD SCI USA | 10.1073/pnas.0608008103 | 197 | 10.37 | 4.48 |
| KONG GYX, 2009, J GLAUCOMA | 10.1097/IJG.0b013e318181284f | 192 | 12 | 1.05 |
| STEINKRAUS KA, 2008, AGING CELL | 10.1111/j.1474-9726.2008.00385.x | 190 | 11.18 | 1.89 |
| MCGRATTAN AM, 2019, CURR NUTR REP | 10.1007/s13668-019-0271-4 | 185 | 30.83 | 4.14 |
| MATTSON MP, 2000, BRAIN RES | 10.1016/S0006-8993(00)02790-6 | 185 | 7.4 | 1.09 |
| LIU S, 2020, MOL NEUROBIOL | 10.1007/s12035-020-02073-3 | 182 | 36.4 | 5.59 |
| ABDOLMALEKY HM, 2004, AM J MED GENET B | 10.1002/ajmg.b.20142 | 174 | 8.29 | 2.06 |
| LAU FC, 2005, NEUROBIOL AGING | 10.1016/j.neurobiolaging.2005.08.007 | 172 | 8.6 | 1.69 |
| DING JD, 2011, P NATL ACAD SCI USA | 10.1073/pnas.1100901108 | 169 | 12.07 | 1.97 |
| LINDEFELDT M, 2019, NPJ BIOFILMS MICROBI | 10.1038/s41522-018-0073-2 | 150 | 25 | 3.35 |
| FITZ NF, 2010, J NEUROSCI | 10.1523/JNEUROSCI.1051-10.2010 | 146 | 9.73 | 1.67 |
| LE DOUCE J, 2020, CELL METAB | 10.1016/j.cmet.2020.02.004 | 143 | 28.6 | 4.39 |
| CUNNANE SC, 2016, FRONT MOL NEUROSCI | 10.3389/fnmol.2016.00053 | 140 | 15.56 | 3.55 |
| MATTSON MP, 2004, ANN NY ACAD SCI | 10.1196/annals.1306.004 | 129 | 6.14 | 1.52 |
| ZHU HY, 1999, BRAIN RES | 10.1016/S0006-8993(99)01827-2 | 128 | 4.92 | 0.63 |
| GILLETTE-GUYONNET S, 2013, BRIT J CLIN PHARMACO | 10.1111/bcp.12058 | 127 | 10.58 | 1.83 |
| NETH BJ, 2020, NEUROBIOL AGING | 10.1016/j.neurobiolaging.2019.09.015 | 125 | 25 | 3.84 |
| CAI WJ, 2014, P NATL ACAD SCI USA | 10.1073/pnas.1316013111 | 125 | 11.36 | 3.05 |
| O'BRIEN JT, 2017, J PSYCHOPHARMACOL | 10.1177/0269881116680924 | 121 | 15.13 | 3.37 |
| YAO J, 2011, PLOS ONE | 10.1371/journal.pone.0021788 | 121 | 8.64 | 1.41 |
| EDWARDS C, 2014, AGING-US | 10.18632/aging.100683 | 120 | 10.91 | 2.93 |
| LOREN DJ, 2005, PEDIATR RES | 10.1203/01.PDR.0000157722.07810.15 | 119 | 5.95 | 1.17 |
| ROTHMAN SM, 2012, ANN NY ACAD SCI | 10.1111/j.1749-6632.2012. 06525.x | 116 | 8.92 | 2.5 |

| Notes: TC: Total Citations. |
| --- |

**Table S2.** Publication and Citation Profiles of Leading Countries.

| **Country** | **Articles** | **Freq** | **SCP** | **MCP** | **MCP-Ratio** | **TP** | **TP-rank** | **TC** | **TC-rank** | **Average Citations** |
| --- | --- | --- | --- | --- | --- | --- | --- | --- | --- | --- |
| USA | 223 | 0.403 | 185 | 38 | 0.170 | 889 | 1 | 15489 | 1 | 69.5 |
| CHINA | 59 | 0.106 | 49 | 10 | 0.169 | 232 | 2 | 1570 | 3 | 26.6 |
| SPAIN | 27 | 0.049 | 17 | 10 | 0.370 | 147 | 3 | 894 | 5 | 33.1 |
| ITALY | 23 | 0.042 | 17 | 6 | 0.261 | 82 | 7 | 601 | 7 | 26.1 |
| FRANCE | 22 | 0.040 | 13 | 9 | 0.409 | 94 | 4 | 977 | 4 | 44.4 |
| UNITED KINGDOM | 19 | 0.034 | 7 | 12 | 0.632 | 83 | 6 | 613 | 6 | 32.3 |
| AUSTRALIA | 16 | 0.029 | 9 | 7 | 0.438 | 92 | 5 | 567 | 9 | 35.4 |
| CANADA | 16 | 0.029 | 9 | 7 | 0.438 | 67 | 8 | 590 | 8 | 36.9 |
| JAPAN | 14 | 0.025 | 13 | 1 | 0.071 | 53 | 9 | 345 | 12 | 24.6 |
| GERMANY | 11 | 0.020 | 7 | 4 | 0.364 | 43 | 10 | 360 | 11 | 32.7 |
| INDIA | 11 | 0.020 | 9 | 2 | 0.182 | 36 | 13 | 2579 | 2 | 234.5 |
| BRAZIL | 10 | 0.018 | 8 | 2 | 0.200 | 40 | 11 | 239 | 14 | 23.9 |
| SOUTH KOREA | 9 | 0.016 | 9 | 0 | 0.000 | 33 | 15 | 100 | 22 | 11.1 |
| TURKEY | 8 | 0.014 | 5 | 3 | 0.375 | 32 | 16 | 148 | 18 | 18.5 |
| NETHERLANDS | 7 | 0.013 | 2 | 5 | 0.714 | 36 | 14 | 526 | 10 | 75.1 |
| IRAN | 6 | 0.011 | 4 | 2 | 0.333 | 24 | 19 | 86 | 25 | 14.3 |
| ISRAEL | 5 | 0.009 | 3 | 2 | 0.400 | 19 | 20 | 63 | 28 | 12.6 |
| MEXICO | 5 | 0.009 | 4 | 1 | 0.200 | 16 | 21 | 65 | 27 | 13 |
| EGYPT | 4 | 0.007 | 2 | 2 | 0.500 | 10 | 24 | 72 | 26 | 18 |
| THAILAND | 4 | 0.007 | 4 | 0 | 0.000 | 10 | 26 | 112 | 21 | 28 |

Notes: Articles: Publications of Corresponding Authors only. Freq: Frequence of Total Publications. SCP: Single Country publications. MCP: Multiple Country Publications. MCP_Ratio: Proportion of Multiple Country Publications. TP: Total Publications. TP_rank: Rank of Total Publications. TC: Total Citations. TC_rank: Rank of Total Citations. Average Citations: The average number of citations per publication.

**Table S3**. Publication and Citation Profiles of High-Impact Authors.

| **Author** | **H-index** | **G-index** | **M-index** | **PY-start** | **TP** | **TP-Frac** | **TP-rank** | **TC** | **TC-rank** |
| --- | --- | --- | --- | --- | --- | --- | --- | --- | --- |
| MATTSON MP | 25 | 27 | 0.962 | 1999 | 27 | 12.30 | 1 | 4469 | 1 |
| VELLAS B | 10 | 10 | 0.500 | 2005 | 10 | 1.49 | 2 | 780 | 3 |
| ANDRIEU S | 7 | 8 | 0.292 | 2001 | 8 | 0.74 | 3 | 275 | 10 |
| GILLETTE-GUYONNET S | 6 | 6 | 0.300 | 2005 | 6 | 1.47 | 4 | 334 | 8 |
| CORTES F | 5 | 5 | 0.250 | 2005 | 5 | 0.88 | 5 | 154 | 17 |
| ISAACSON RS | 5 | 5 | 0.417 | 2013 | 5 | 0.68 | 6 | 307 | 9 |
| MORRIS MC | 5 | 5 | 0.500 | 2015 | 5 | 1.33 | 9 | 264 | 12 |
| PARK SK | 5 | 5 | 0.625 | 2017 | 5 | 2.08 | 11 | 75 | 23 |
| ANSTEY KJ | 4 | 4 | 0.571 | 2018 | 4 | 0.38 | 14 | 102 | 20 |
| BARNES LL | 4 | 4 | 0.308 | 2012 | 4 | 0.42 | 15 | 171 | 15 |
| GUO ZH | 4 | 4 | 0.160 | 2000 | 4 | 1.00 | 16 | 644 | 6 |
| LAHIRI DK | 4 | 4 | 0.190 | 2004 | 4 | 1.07 | 17 | 152 | 18 |
| LEE J | 4 | 4 | 0.160 | 2000 | 4 | 0.97 | 18 | 970 | 2 |
| PIKE CJ | 4 | 4 | 0.333 | 2013 | 4 | 1.42 | 20 | 180 | 14 |
| VELAZQUEZ R | 4 | 4 | 0.444 | 2016 | 4 | 0.52 | 21 | 155 | 16 |
| WANG J | 4 | 4 | 0.200 | 2005 | 4 | 0.49 | 22 | 654 | 5 |
| BRINTON RD | 3 | 3 | 0.214 | 2011 | 3 | 0.37 | 23 | 201 | 13 |
| CALON F | 3 | 3 | 0.214 | 2011 | 3 | 1.23 | 24 | 74 | 24 |
| CANTET C | 3 | 3 | 0.150 | 2005 | 3 | 0.54 | 26 | 115 | 19 |
| COX KL | 3 | 3 | 0.429 | 2018 | 3 | 0.22 | 28 | 78 | 22 |

Notes: H-index: The index measures both the productivity and citation impact of the author’s publications. G-index: The index evaluates the cumulative impact of the author’s most highly cited publications. M-index: The index is the H-index divided by the number of years since the authors’s first publication. TP: Total Publications. TP_rank: Rank of Total Publications. TC: Total Citations. TC_rank: Rank of Total Citations. Average Citations: The average number of citations per publication. PY_start: Publication Year Start, indicating the year the journal started publication.

**Table S4**. Bibliometric Indicators of High-Impact Journals.

| **Journal** | **H-index** | **IF 2023** | **JCR 2023** | **TP** | **TP-rank** | **TC** | **TC-rank** | **PY-start** |
| --- | --- | --- | --- | --- | --- | --- | --- | --- |
| JOURNAL OF ALZHEIMERS DISEASE | 16 | 3.4 | 2 | 34 | 1 | 842 | 1 | 2004 |
| JOURNAL OF NUTRITION HEALTH & AGING | 12 | 4.3 | 1 | 15 | 2 | 228 | 25 | 2005 |
| PLOS ONE | 12 | 2.9 | 1 | 12 | 3 | 558 | 7 | 2009 |
| CURRENT ALZHEIMER RESEARCH | 10 | 1.8 | 4 | 11 | 4 | 132 | 50 | 2010 |
| NEUROBIOLOGY OF AGING | 9 | 3.7 | 2 | 11 | 5 | 684 | 5 | 2002 |
| ALZHEIMERS & DEMENTIA | 7 | 13.0 | 1 | 7 | 7 | 441 | 11 | 2008 |
| MOLECULAR NEUROBIOLOGY | 7 | 4.6 | 1 | 7 | 10 | 140 | 46 | 2010 |
| BRAIN RESEARCH | 6 | 2.7 | 3 | 7 | 8 | 326 | 15 | 1999 |
| INTERNATIONAL JOURNAL OF MOLECULAR SCIENCES | 6 | 4.9 | 2 | 7 | 9 | 160 | 39 | 2019 |
| NEUROLOGY | 6 | 7.7 | 1 | 6 | 16 | 781 | 3 | 1998 |
| JOURNAL OF THE AMERICAN MEDICAL DIRECTORS ASSOCIATION | 5 | 4.2 | 2 | 5 | 17 | 66 | 110 | 2009 |
| MEDICAL HYPOTHESES | 5 | 2.1 | 3 | 6 | 15 | 39 | 175 | 2004 |
| NEUROBIOLOGY OF DISEASE | 5 | 5.1 | 1 | 5 | 18 | 217 | 28 | 2007 |
| NUTRIENTS | 5 | 4.8 | 1 | 10 | 6 | 252 | 22 | 2020 |
| PROCEEDINGS OF THE NATIONAL ACADEMY OF SCIENCES OF THE UNITED STATES OF AMERICA | 5 | 9.4 | 1 | 5 | 19 | 792 | 2 | 2006 |
| SCIENTIFIC REPORTS | 5 | 3.8 | 1 | 7 | 11 | 206 | 30 | 2017 |
| AGING CELL | 4 | 8.0 | 1 | 6 | 12 | 204 | 31 | 2005 |
| ALZHEIMER DISEASE & ASSOCIATED DISORDERS | 4 | 1.8 | 3 | 4 | 21 | 104 | 63 | 2001 |
| CURRENT OPINION IN CLINICAL NUTRITION AND METABOLIC CARE | 4 | 3.0 | 2 | 4 | 23 | 34 | 210 | 2008 |
| FASEB JOURNAL | 4 | 4.4 | 2 | 4 | 24 | 196 | 32 | 2004 |

Notes: H-index: The index measures both the productivity and citation impact of the publications. IF: Impact Factor, indicating the average number of citations to recent articles published in the journal. JCR: The quartile ranking of the journal in the Journal Citation Reports, indicating the journal's ranking relative to others in the same field (Q1: top 25%, Q2: 25%-50%, Q3: 50%-75%, Q4: bottom 25%). TP: Total Publications. TP-rank: Rank of Total Publications. TC: Total Citations. TC-rank: Rank of Total Citations. PY-start: Publication Year Start, indicating the year the journal started publication.
